# *Drosophila* Ge-1 Promotes P Body Formation and *oskar* mRNA Localization

**DOI:** 10.1371/journal.pone.0020612

**Published:** 2011-05-31

**Authors:** Shih-Jung Fan, Virginie Marchand, Anne Ephrussi

**Affiliations:** Developmental Biology Unit, European Molecular Biology Laboratory, Heidelberg, Germany; Skirball Institute of Biomolecular Medicine - New York University Medical Center, United States of America

## Abstract

mRNA localization coupled with translational control is a widespread and conserved strategy that allows the localized production of proteins within eukaryotic cells. In *Drosophila*, *oskar* (*osk*) mRNA localization and translation at the posterior pole of the oocyte are essential for proper patterning of the embryo. Several P body components are involved in *osk* mRNA localization and translational repression, suggesting a link between P bodies and *osk* RNPs. In cultured mammalian cells, Ge-1 protein is required for P body formation. Combining genetic, biochemical and immunohistochemical approaches, we show that, *in vivo*, *Drosophila Ge-1* (*dGe-1*) is an essential gene encoding a P body component that promotes formation of these structures in the germline. dGe-1 partially colocalizes with *osk* mRNA and is required for *osk* RNP integrity. Our analysis reveals that although under normal conditions *dGe-1* function is not essential for *osk* mRNA localization, it becomes critical when other components of the localization machinery, such as *staufen*, *Drosophila decapping protein 1* and *barentsz* are limiting. Our findings suggest an important role of dGe-1 in optimization of the *osk* mRNA localization process required for patterning the *Drosophila* embryo.

## Introduction

*oskar* (*osk*) (FlyBase: CG10901) mRNA localization and translation at the posterior pole of the *Drosophila* oocyte are essential for antero-posterior patterning of the embryo, their failure resulting in embryos lacking an abdomen and germline, the so-called posterior group phenotype [1], [2]. During oogenesis, *osk* is transcribed in the nurse cells and, upon splicing, begins to assemble into ribonucleoprotein (RNP) complexes that are transported into the cytoplasm and through the actin-rich ring canals of the nurse cells into their sibling cell, the oocyte, where ultimately the RNA is localized at the posterior pole [3]. Through years of genetic and biochemical analysis, proteins involved in *osk* post-transcriptional regulation have been identified. These include, *Drosophila* decapping protein 1 (dDcp1) (FlyBase: CG11183) and Me31B (FlyBase: CG4916), whose yeast and mammalian counterparts are components of cytoplasmic granules named Processing bodies (P bodies) [4], [5], [6].

P bodies have been described in many eukaryotes and consist of aggregates of translationally inactive RNPs [7], [8]. The number and size of these dynamic structures depends on the availability of mRNAs not associated with the translational machinery [7], [9], [10]. Proteins of the mRNA degradation machinery, such as Dcp1 and Dhh1, and translational repressors, such as RAP55 and 4E-T, are enriched in P bodies [7], [8]. Although P bodies are conserved structures, their disruption seems to affect neither mRNA decay nor translational repression [6], [11]. It has therefore been proposed that the role of P bodies might be to compartmentalize mRNA decay and translation repression, possibly enhancing the efficiency of these processes [7].

In yeast, the Yjef-N dimerization domain and the prion-like Glutamine/Asparagine (Q/N)-rich domain of two P body components, Edc3 and Lsm4, respectively, are required for P body assembly [11], [12], suggesting that P body formation might be a self-assembly process [7], [13]. However, in higher eukaryotic cells the Yjef-N domain of Edc3 plays only a minor role in P body assembly [14] and the Q/N domain of yeast Lsm4 is not found in its eukaryotic homologues, suggesting that Lsm4 either performs its function by a different mechanism or does not promote P body formation in these organisms. Interestingly, a conserved protein with no homologue in yeast, Ge-1, contains at its C-terminus a Q/N domain that promotes oligomerization of the protein *in vitro*, and at its N-terminus a WD40 repeat, a domain often involved in protein-protein interactions [11], [15], [16], [17], [18]. Ge-1 can associate with Dcp1 *in vitro* to enhance decapping activity [15], [16] and *Arabidopsis* Ge-1 is involved in postembryonic development, by regulating the decapping process [16]. In different eukaryotic cells, *Ge-1* knockdown causes P bodies to disappear [15], [16], [17], [19]. While it is clear that Ge-1 plays a critical role in P body formation in cells, its *in vivo* functions in metazoans remain largely unexplored.

In this manuscript, we describe our experiments aimed at determining the importance and role of Ge-1 in a living organism, *Drosophila melanogaster*. We show that *Drosophila Ge-1 (dGe-1)* (FlyBase: CG6181) is an essential gene that encodes a genuine P body component critical for P body integrity in the fly germline. Our *in vivo* analysis of dGe-1 function in the germline reveals that dGe-1 promotes the assembly of *osk* transport RNPs and colflaborates with the *osk* RNP components Staufen (Stau) (FlyBase: CG5753), Barentsz (Btz) (FlyBase: CG12878) and dDcp1 in *osk* mRNA localization and *Drosophila* embryonic patterning.

## Results and Discussion

### *dGe-1* is a lethal gene in *Drosophila*

**Figure 1:**
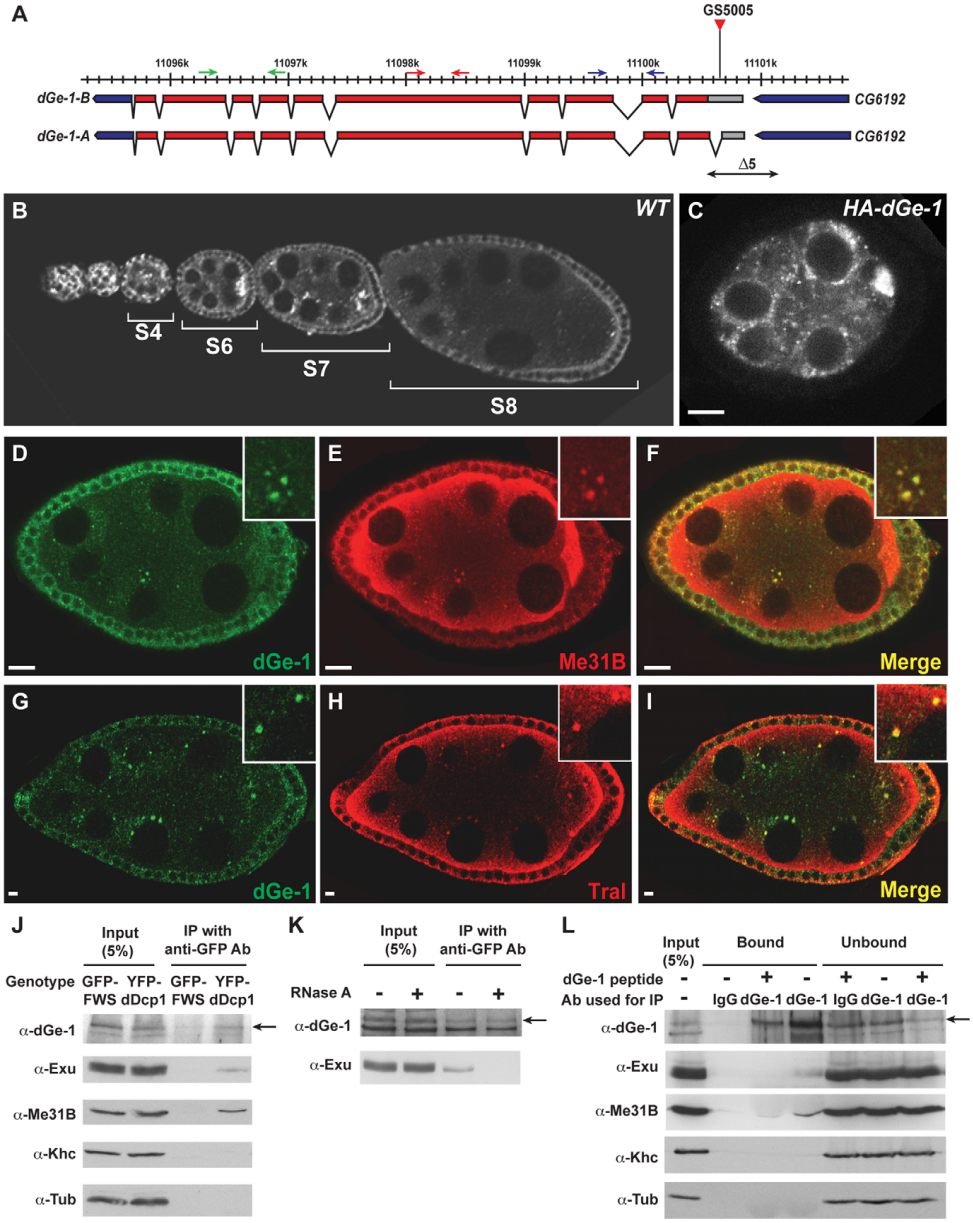
dGe-1 is a P body component in the *Drosophila* germline. (A) Scheme representing the *dGe-1* locus (polytene band 32D3). Numbers indicate genomic positions along chromosome 2L. 5′UTR, coding region, and 3′UTR: grey, red and blue boxes, respectively. The two *dGe-1* transcripts, *dGe-1-A* and *dGe-1-B*, the insertion site of the P-element transposon (GS5005, red triangle) used for generation of *dGe-1* deletions, and the region deleted in *dGe-1^Δ5^* (double-headed arrow) are represented. Positions of primers used for RT-PCR (Figure S1B) are indicated (blue, red, green arrows). (B) Distribution of dGe-1 protein in *wt* egg-chambers during early and mid-oogenesis. Immunodetection of dGe-1 using rat anti-dGe-1 antibody. (C) HA-dGe1 protein distribution in *wt* egg-chamber at stage 6 (S6). HA-dGe-1 detected using mouse anti-HA antibody. (D–I) Colocalization of dGe-1 protein and two P body components. Double-staining of two *wt* S7 egg-chambers using rat anti-dGe1 (green, D and G) and rabbit anti-Me31B (red, E) or rabbit anti-Tral (red, H) antibodies. Overlays (F and I). (J) Association of dGe-1 and dDcp1 in ovarian extract. Western blot of anti-GFP immunoprecipitates of *GFP-FWS* and *YFP-dDcp1* ovaries. Western blot probed with rabbit anti-dGe-1, anti-Exu and anti-Khc, and mouse anti-Me31B and anti-Tub antibodies. dGe-1 is indicated with an arrow (see legend to Figure 2A). (K) RNA-independent association of dGe-1 and dDcp1. Western blot of anti-GFP immunoprecipitates from *YFP-dDcp1* ovarian extract with or without RNase A treatment prior to immunoprecipitation, probed with rabbit anti-dGe-1 and anti-Exu antibodies. dGe-1 is indicated with an arrow. (L) Exu and Me31B associate with dGe-1 in the ovary. Endogeneous dGe-1 protein was immunoprecipitated from *wt* ovarian extract and bound and soluble fractions were subjected to western blot analysis. Before immunoprecipitation, rabbit anti-dGe-1 was pre-incubated or not with dGe-1 blocking peptide. Western blot probed with rabbit anti-dGe-1, anti-Exu and anti-Khc, and mouse anti-Me31B and anti-Tub antibodies. dGe-1 is indicated with an arrow. Bar, 10 µm.

The *dGe-1* locus encodes two transcripts, *dGe-1-A* and *dGe-1-B*, which differ only in their 5′ UTRs and are transcribed during oogenesis (Figure 1A and S1A). To address the function of *dGe-1* during oogenesis, we generated deletion mutants by imprecise excision of a P element in the locus (Figure 1A). One of these, *dGe-1^Δ5^* is a recessive lethal mutation whose lethality was rescued by transgenic expression of a *dGe-1-B* cDNA, proving is a lethal *dGe-1* allele.

**Figure 2:**
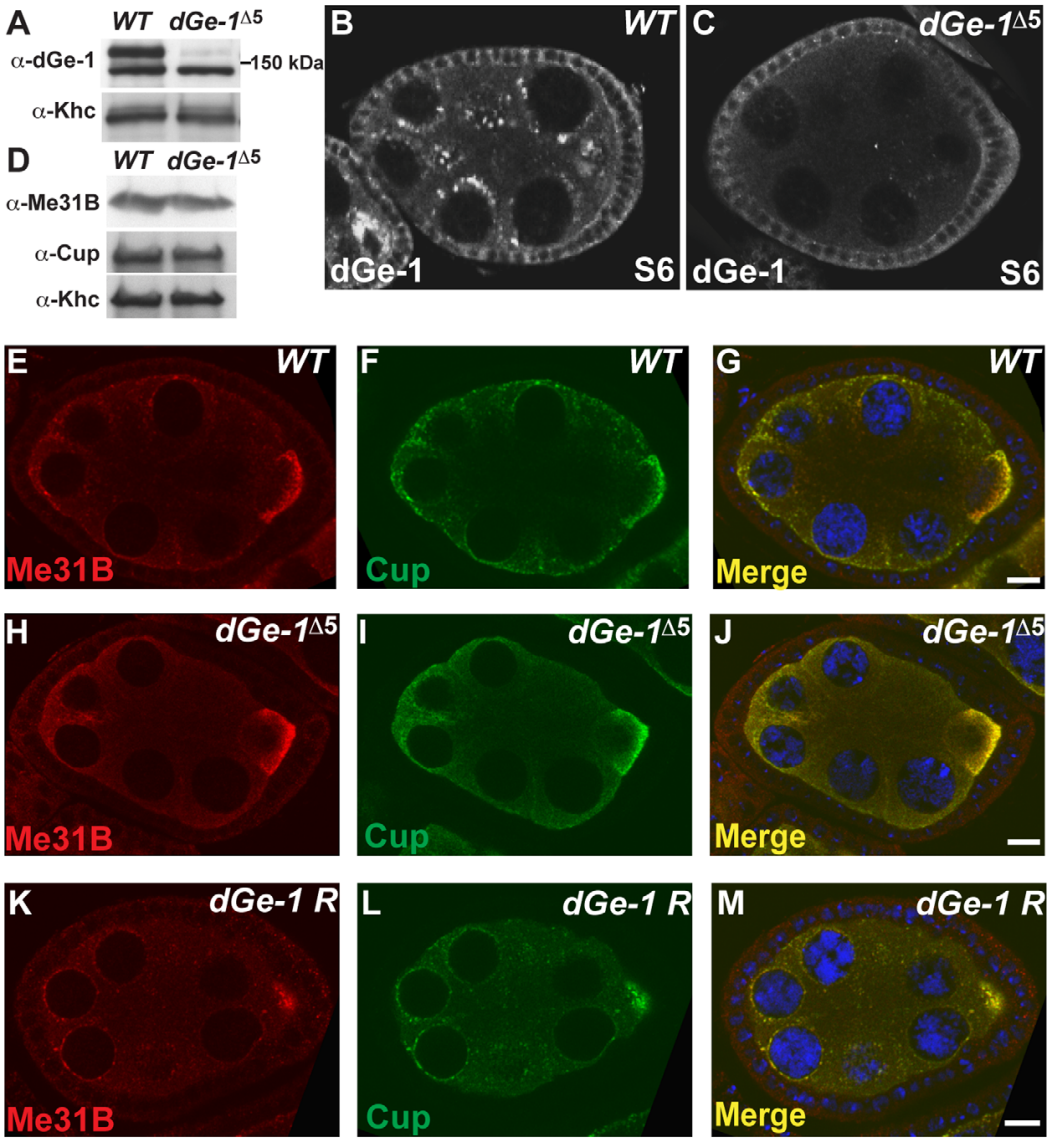
dGe-1 promotes P body formation in the fly germline. (A) dGe-1 protein levels are strongly reduced in *dGe-1^Δ5^ GLC*. Western blot of dGe-1 protein in extract of 0-2 h *wt* or *dGe-1^Δ5^ GLC* embryos probed with rat anti-dGe-1 antibodies. The band just above 150 kDa, which is dramatically reduced in the mutant extract, represents dGe-1 protein. Khc protein serves as a loading control. (B-C) dGe-1 signal is absent from the nurse cells and oocyte of *dGe-1^Δ5^ GLC*. Immunodetection of dGe-1 protein in *wt* (B) and *dGe-1^Δ5^ GLC* (C) ovaries using rat anti-dGe-1 antiserum. (D) Western blot analysis of ovarian extracts from *wt* and *dGe-1^Δ5^ GLC* ovaries, probed with mouse anti-Me31B and anti-Cup antibodies and rabbit anti-Khc antibody. Khc serves as a loading control. (E–K) Immunostaining of *wt* (E–G), *dGe-1^Δ5^ GLC* (H–J) and *dGe-1^Δ5^/dGe-1^Δ5^*; *dGe-1* Rescue (*dGe-1 R*) (K–M) S7 egg-chambers using a rabbit anti-Me31B antibody (E, H, K) or a mouse anti-Cup antibody (F, I, L). Overlays are shown in G, J and M. Bar, 10 µm.

Analysis of *dGe-1^Δ5^* ovaries requires generation of homozygous mutant *dGe-1^Δ5^* germline clones (*GLC*) [20]. *dGe-1* mRNA levels are dramatically decreased in *dGe-1^Δ5^ GLC* ovaries and no trunfcated *dGe-1* transcript produced from sequences downstream of the deleted region was detected (Figure S1B). Western blots of extracts of young embryos probed with anti-dGe-1 antibody showed two bands of around 150 kDa, the upper of which was dramatically reduced in extracts of embryos derived from *dGe-1^Δ5^ GLC*, indicating that it represents dGe-1 protein (Figure 2A). Hence, *dGe-1^Δ5^* is a strong hypomorphic allele of *dGe-1* both at the genetic and the molecular levels.

### dGe-1 is a P body component in the *Drosophila* germline

dGe-1 is detected in the cytoplasm of the nurse cells and oocyte in *wild-type* (*wt*) ovaries, from early oogenesis onward (Figure 1B), but is absent from these cells in *dGe-1^Δ5^ GLC* (Figure 2B–C). dGe-1 is also expressed in the follicle cells surrounding the germline cyst. Both endogenous dGe-1 and transgenic HA-tagged dGe-1 protein are detected in puncta reminiscent of P bodies in the nurse cells (Figure 2B and 1C). Indeed, two P body markers, Me31B and Trailer hitch (Tral, *Drosophila* homologue of RAP55) (FlyBase: CG10686) [6] colocalize with dGe-1 in puncta (Figure 1D–I). The colocalization of dGe-1 protein with two major P body components suggested that dGe-1 might be a P body component in *Drosophila* ovaries.

Ge-1 has been shown to associate with Dcp1 in mammalian cells and *Arabidopsis* [15], [16]. dGe-1, as well as Me31B and Exu (FlyBase: CG8994), a known dDcp1 interactor [4], are selectively enriched in anti-GFP immunoprecipitates from ovarian extract of YFP-dDcp1 expressing flies (Figure 1J), but not from FWS-GFP expressing flies (Four Way Stop (FWS) is a Golgi protein with no known role in mRNA degradation and translation) [21]. Similarly, Me31B and Exu are selectively enriched in dGe-1 immunoprecipitates from *wt* ovarian extract (Figure 1L). Treatment of the YFP-dDcp1 extract with RNase A prior to immunoprecipitation did not affect the association of dGe-1 with YFP-dDcp1, whereas the association of Exu with dDcp1 is RNA-dependent [4] and was lost (Figure 1K). It therefore appears that, in the ovary, dGe-1 and dDcp1 associate via protein-protein interaction. Taken together, these results indicate that dGe-1, dDcp1, Me31B and Exu are constituents of a shared RNP structure and that dGe-1 is a P body component in the *Drosophila* germline.

### dGe-1 promotes P body formation in the *Drosophila* germline

To determine if dGe-1 is required for P body formation in the *Drosophila* germline, we examined the distribution of the P body markers, Me31B and Cup (the *Drosophila* functional homologue of 4E–T) (FlyBase: CG11181) [6], [22], [23] in *dGe-1^Δ5^ GLC* ovaries. The overall amounts of these proteins are similar in *dGe-1^Δ5^ GLC* and *wt* extracts (Figure 2D). However the distribution of these proteins is severely affected in the mutant, where they are no longer detected in granules and are uniformly distributed in the nurse cells (compare Figure 2H–J to Figure 2E–G). The granular distribution of Me31B and Cup in *dGe-1^Δ5^ GLC* egg-chambers is restored by transgenic expression of a *dGe-1-B* cDNA (Figure 2K–M). Therefore, as in cultured cells [15], [16], [17], [19], dGe-1 is involved in P body formation in the *Drosophila* germline.

### *dGe-1* is involved in *osk* mRNA localization and embryonic patterning

To understand the role of dGe-1 in mRNA regulation *in vivo*, we evaluated its association with RNAs in the ovary. All RNAs tested, including the localized RNAs *gurken* (*grk*) (FlyBase: CG17610) [24], *bicoid* (*bcd*) (FlyBase: CG1034) [25] and *osk* [26], [27], and the abundant RNAs, *tubulin* (*tub*) (FlyBase: CG8308) and *ribosomal protein 49* (*rp49*) (FlyBase: CG7939), were enriched upon dGe-1 immunoprecipitation (Figure S2A), consistent with a general role of P bodies in mRNA regulation.

We next tested if dGe-1 has a role in *grk*, *bcd* or *osk* mRNA localization or translational control. *In situ* hybridization revealed that *bcd* and *grk* localize normally in *dGe-1^Δ5^ GLC* (Figure S2B–D, F), although in some oocytes the amount of Grk protein in the antero-dorsal corner appears reduced (Figure S2E and G). Western blot analysis confirmed that Grk protein levels are reduced in *dGe-1^Δ5^ GLC* ovaries and embryos (Figure S2H), although no obvious difference in *grk* mRNA levels was observed by qRT-PCR in *dGe-1^Δ5^ GLC* compared to *wt* ovarian extracts (data not shown). Consistent with this, around 17% of eggs produced from *dGe-1^Δ5^ GLC* develop defective dorsal appendages. Importantly, both Grk protein levels and dorsal appendage defects can be rescued by transgenic expression of a *dGe-1-B* cDNA, demonstrating an involvement, either direct or indirect, of *dGe-1* in Grk expression.

**Figure 3:**
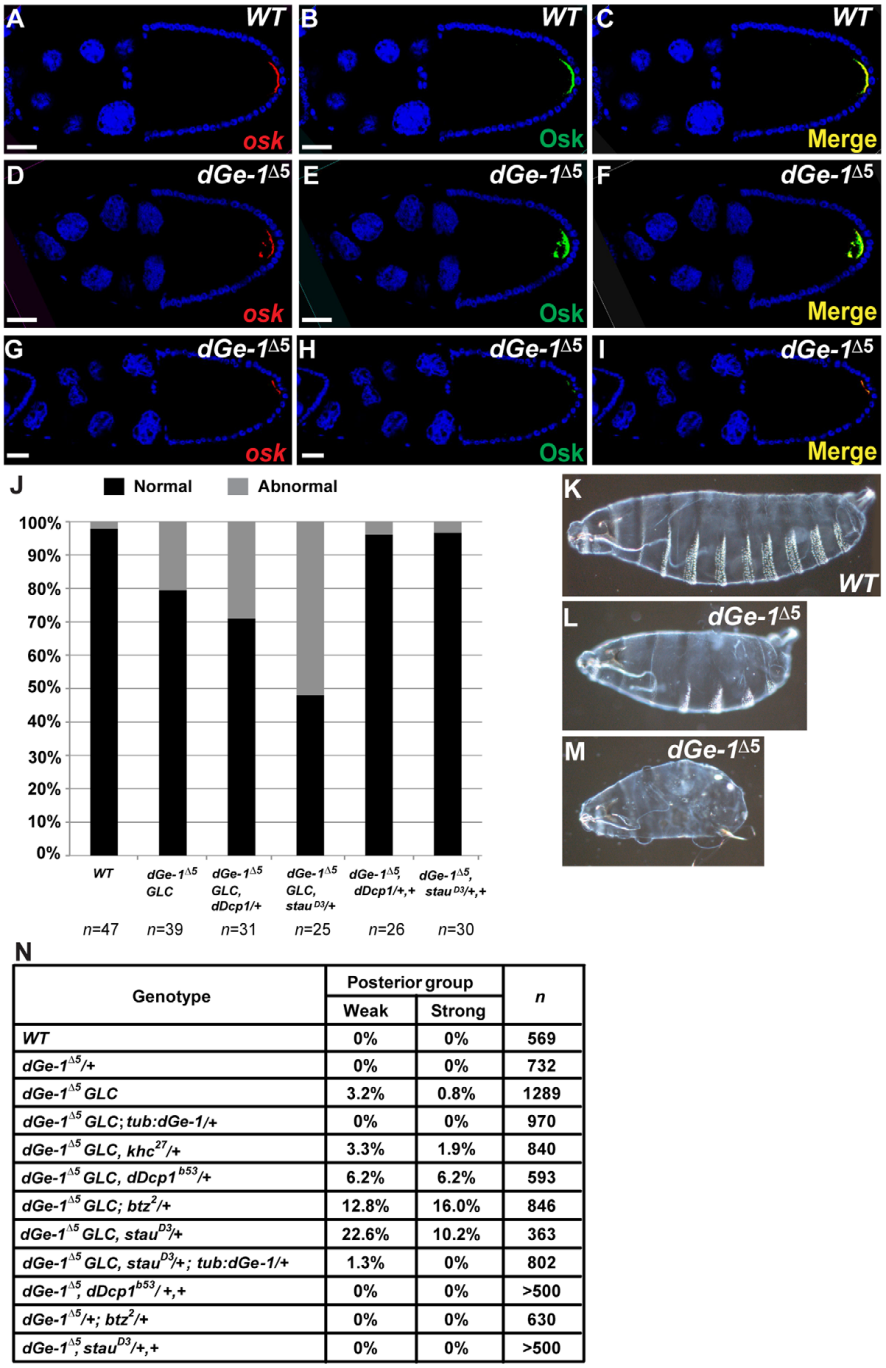
dGe-1 cooperates with Stau and dDcp1 in osk mRNA localization and embryonic patterning. (A–I) Simultaneous detection of *osk* mRNA (red) and protein (green) by FISH coupled with immunodetection using a rabbit anti-Osk antibody. *osk* mRNA (A) and protein (B) colocalize in a posterior crescent at the posterior pole of *wt* oocytes at S10. (D–I) Examples of the aberrant distribution of *osk* mRNA and protein in *dGe-1^Δ5^ GLC* egg-chambers. Overlays of *osk* mRNA and Osk protein signals (C, F, I). DNA stained with 4,6-diamidino-2-phenylindole (DAPI) (blue). (J) Quantification (%) of *osk* mRNA localization patterns in S10 egg-chambers in different genetic backgrounds. Normal and abnormal *osk* mRNA localization are represented as black and grey bars, respectively. *n* represents the number of embryos analyzed. (K–M) Loss of maternal *dGe-1* causes aberrant cuticle patterning in some embryos derived from *dGe-1^Δ5^ GLC*. Lateral view of embryos oriented anterior to the left, ventral side down. (K) In a *wt* embryo, posterior structures represented by the abdominal denticle belts are clearly visible along the ventral side. In a portion of embryos derived from *dGe-1^Δ5^ GLC*, several denticle belts are missing (L) and, in extreme cases, all are absent (M). (N) Quantification of posterior group phenotypes produced in different genetic backgrounds. Embryos lacking at least one (weak) or all (strong) abdominal denticle belts were quantified. *n* represents the number of embryos analyzed. Bar, 50 µm.

In addition, in contrast to *wt* oocytes (Figure 3A–C), a significant proportion of *dGe-1^Δ5^ GLC* oocytes displays defective *osk* mRNA localization at the posterior pole at stages 9 and 10 (S9, S10) (Figure 3D, 3G and 3J and Figure S3A). This mislocalization, which is rescued by transgenic expression of a *dGe-1-B* cDNA (Figure S3B–D), is paralleled by defects in Osk protein localization (Figure 3E and 3H), and Osk protein levels are reduced in *dGe-1^Δ5^ GLC* ovaries (Figure S3E).

Around 55% of the eggs produced by *dGe-1^Δ5^ GLC* females develop into embryos and hatch. Among the 45% of the eggs produced by *dGe-1^Δ5^ GLC* females that fail to hatch, half (23% of the total) show a normal cuticle. Among the other half, about 16%, presumably unfertilized, develop no cuticle (data not shown). Around 3.2% develop into embryos displaying a weak, and 0.8% a strong posterior group phenotype (Figure 3K–N), consistent with impairment of *osk* mRNA localization and translation. Another 2.7% develop into embryos showing a variety of patterning defects (such as head open). Importantly, the posterior patterning of *dGe-1^Δ5^ GLC* embryos is rescued by expression of a *dGe-1-B* cDNA in the maternal germline (Figure 3N). These results demonstrate that dGe-1 has a role in abdominal patterning in *Drosophila*.

### *dGe-1* interacts genetically with genes involved in *osk* mRNA localization

To further investigate the involvement of *dGe-1* in *osk* localization, we tested its interaction with other genes involved in the process. Kinesin heavy chain (Khc) (FlyBase: CG7765) is essential for *osk* transport [28]. Removal of one *wt* copy of *khc* did not enhance posterior patterning defects in *dGe-1^Δ5^ GLC* embryos (Figure 3N), suggesting that *dGe-1* and *khc* are involved in distinct aspects of *osk* mRNA localization. Removal of one *wt* copy of *dDcp1*, *btz*, or *stau*, whose protein products colocalize with *osk* mRNA and are required for its localization [4], [29], [30], increased the penetrance of the posterior group phenotype among *dGe-1^Δ5^ GLC*-derived embryos from 4% to 12.4, 28.8 and 32.8%, respectively (Figure 3N). In contrast, embryos produced by females lacking a copy of *dDcp1*, *btz or stau*, but heterozygous for *dGe-1* displayed normal abdominal patterning (Figure 3N), highlighting the requirement for *dGe-1* in this process. Confirming this, the posterior patterning defects of embryos developing from *dGe-1^Δ5^ GLC* heterozygous for *stau* were fully rescued by a *dGe-1-B* cDNA transgene (Figure 3N).

We also assayed the genetic interaction of *dGe-1* with *osk* RNP components by assaying *osk* mRNA localization directly. Whereas heterozygosity for *dGe-1^Δ5^* and *dDcp1* or *stau* did not cause *osk* mislocalization, loss of both *wt* copies of *dGe-1^Δ5^* in a *dDcp-1* or *stau* heterozygous background significantly increased the percentage of oocytes with abnormal *osk* mRNA localization over that of simple *dGe-1^Δ5^ GLC* (Figure S3A and 3J). Taken together, these results show that *dGe-1* and the *osk* RNP components cooperate in *osk* mRNA localization.

### dGe-1 colocalizes with *osk* mRNA

**Figure 4:**
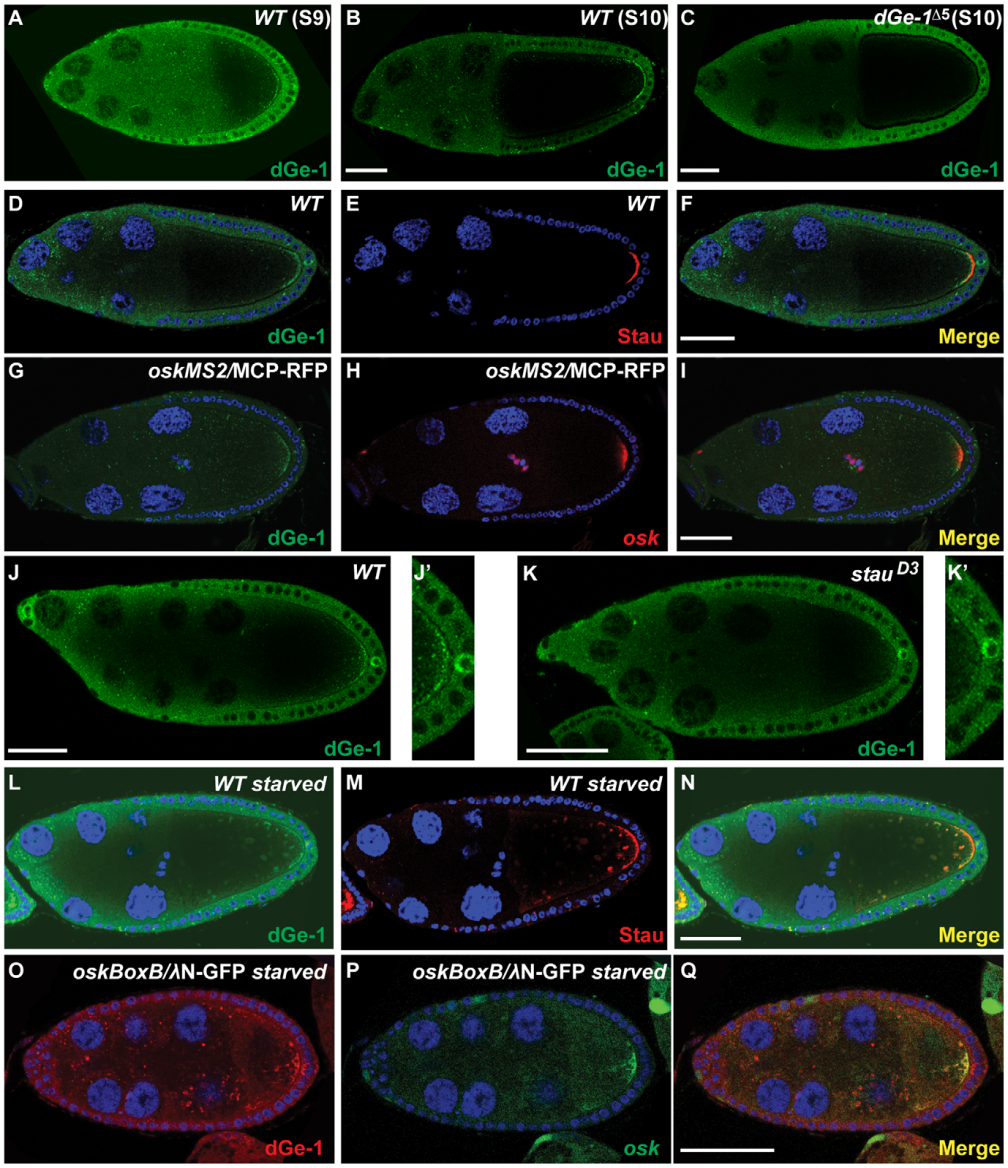
dGe-1 partially colocalizes with *osk* mRNA. (A–C) Immunodetection of dGe-1 protein in *wt* (A, B) and *dGe-1^Δ5^ GLC* (C) ovaries using a rabbit anti-dGe-1 antibody. (A, B) In *wt* ovaries at S9 (A) or S10 (B), dGe-1 protein is enriched at the oocyte posterior pole. (C) The posterior dGe-1 signal observed in *wt* oocytes (B) is greatly reduced in *dGe-1^Δ5^ GLC* oocytes. (D–F) In a *wt* egg-chamber at late S9, dGe-1 enriched at the posterior pole of the oocyte (green, D) and partially colocalizes with Stau protein (red, E). The overlay of the two antibody signals is shown in (F). (G–I) Colocalization of dGe-1 protein (green, G) and *osk* mRNA (red, H) in *wt* S9 oocytes expressing *oskMS2* mRNA and MCP-RFP protein (red, H), to which the RNA is tethered; at S9 *oskMS2* mRNA distribution reflects that of endogenous *osk* mRNA [33]. Overlay of dGe-1 and *oskMS2* mRNA signals shown in (I). (J–K) dGe-1 protein staining in *wt* and *stau^D3^* oocytes. (J, J′) dGe-1 protein is enriched at the posterior pole in *wt* oocytes. (K and K′) In *stau^D3^* mutant oocytes, which fail to localize *osk* mRNA, the amount of dGe-1 at the posterior is dramatically reduced. (L–N) Double immunostaining of dGe-1 and Stau proteins in a nutritionally restricted *wt* egg-chamber. In starved *wt* oocytes, dGe-1 protein (green, L) is present in large particles, where it colocalizes with Stau protein (red, M). Overlay of dGe-1 and Stau stainings (N). (O–Q) Distribution of dGe-1 protein and *osk* mRNA in starved *wt* egg-chambers expressing *oskBoxB* mRNA and λN-GFP protein, to which the RNA is tethered. In nutritionally restricted *wt* oocytes, dGe-1 (red, O) is present in large particles, where it partially colocalizes with *oskBoxB* mRNA (green, P). Overlay of dGe-1 and *osk* mRNA signals shown in (Q). Bar, 50 µm.

In addition to its punctate distribution in the nurse cells, dGe-1 protein is also observed in a crescent at the oocyte posterior during mid-oogenesis (Figure 4A–B). This staining is specific, as it is dramatically reduced in *dGe-1^Δ5^* mutant oocytes (Figure 4C). Moreover, the posterior crescent of dGe-1 overlaps with that of Stau, an *osk* mRNA associated protein, and with *osk* mRNA (Figure 4D–I), suggesting that dGe-1 protein is a component of *osk* RNPs.

To ascertain if dGe-1 is associated with *osk* mRNA *in vivo*, we tested if its distribution in the oocyte depends on *osk* mRNA. In *stau^D3^* oocytes, which fail to localize *osk* mRNA [30], posterior localization of dGe-1 is disrupted (Figure 4J–K). Additionally, the *osk* mRNA and Stau protein aggregates that form upon nutritional restriction of females [31] also contain dGe-1 protein (Figure 4L–Q). Taken together, dGe-1's colocalization with *osk* mRNA and Stau, as well as its dependence on *stau* for posterior localization, suggest that *in vivo* some amount of dGe-1 is associated with *osk* RNPs.

### dGe-1 is required for *osk* RNP integrity

**Figure 5:**
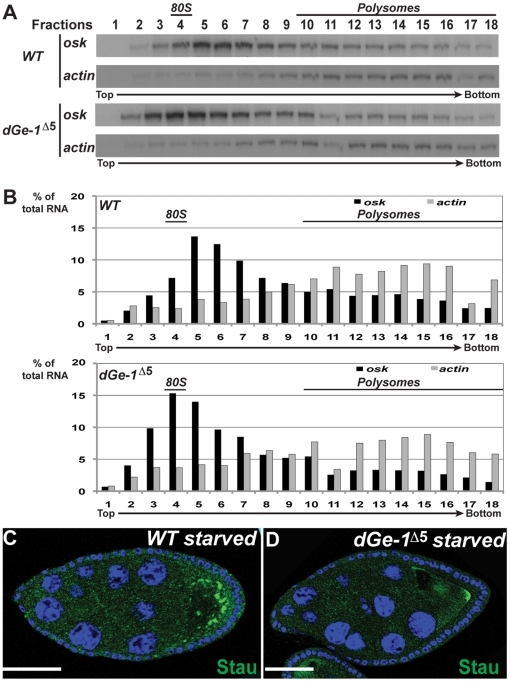
dGe-1 is required for assembly of *osk* RNPs. (A) *Drosophila wt* (upper panel) or *dGe-1^Δ5^ GLC* (lower panel) ovarian extracts treated with cycloheximide and analyzed by centrifugation in a 10–45% sucrose density gradient. *osk* and *actin* mRNAs in the gradient fractions were detected by RNase protection assay. Absorption at 260 nm was measured to situate the monoribosome peak (80S) and polysomes. (B) Quantification of the RNase protection assays presented in (A). The relative levels of *osk* and *actin* mRNAs present in each fraction are indicated as a % of the total levels of both mRNAs present in *wt* (upper panel) and *dGe-1^Δ5^ GLC* (lower panel) ovarian extracts. (C–D) Distribution of Stau protein in starved *wt* (C) or *dGe-1^Δ5^ GLC* (D) ovaries. In nutritionally restricted *wt* oocytes, Stau (green) is present in large particles, which fail to form in starved *dGe-1^Δ5^ GLC* ovaries. Bar, 50 µm.

dGe-1 protein is required for P body formation (Figure 2E–J) and is associated with *osk* RNPs (Figure 4). To determine if dGe-1 might be required for *osk* RNP assembly, we examined the distribution on sucrose gradients of *osk* mRNA in cytoplasmic extracts of *wt* and *dGe-1^Δ5^ GLC* ovaries. In *wt* extracts *osk* mRNA is broadly distributed and enriched in fractions 5 to 7 (Figure 5A–B). In comparison, translationally active *actin* mRNA (FlyBase: CG4027) displays a typical polysome profile, with much of the mRNA enriched in the polysomal fractions (Figure 5A–B, fractions 10 to 18). While in *dGe-1^Δ5^ GLC* extracts *osk* mRNA is also distributed broadly, it is most enriched in fractions 3 to 6 (Figure 5A–B). This shift of *osk* mRNA to the lighter sucrose gradient fractions in *dGe-1^Δ5^* mutant extracts is significant, as no obvious change in the sedimentation profile of *actin* mRNA was observed. Hence, in the *dGe-1^Δ5^* mutant background the size of *osk* RNPs is selectively affected, revealing a role of dGe-1 in their assembly or stability. Consistent with this, large *osk* granules (represented by Stau) induced by nutritional restriction fail to form in *dGe-1^Δ5^ GLC* (Figure 5C–D). In the future, it could be of interest to assess the size of *osk* RNPs in *wt* and *dGe-1* mutant oocytes, for instance by observing their ultrastructure using electron microscopy.

During oogenesis dGe-1 is required for P body formation. This raises the possibility that the mild effect of *dGe-1* on *osk* mRNA localization might be a consequence of P body disruption. It has been shown that upon nutritional restriction, sponge bodies, which are P body-like structures in the *Drosophila* germline that contain many P body components, are enlarged, and interestingly, *osk* RNPs are detected within them [31]. Therefore, a direct interaction between *osk* RNPs and P body-like structures or P bodies themselves may occur, but how this interaction would influence *osk* mRNA localization remains unclear. In the future, it will be interesting to determine the dynamic relationship between P bodies and *osk* RNPs, for instance by live-imaging of shared and distinct components and tracking their movement relative to these two RNP structures.

Alternatively, dGe-1 might act directly in an aspect of *osk* mRNA regulation that is not absolutely essential, such as optimization of the localization process. In support of this hypothesis, our localization and biochemical analyses suggest that, although in the absence of dGe-1 *osk* RNP assembly may be mildly impaired, under normal laboratory conditions small *osk* RNPs can form and are sufficiently functional for mRNA localization and posterior patterning to proceed. However, a strong increase in *osk* localization and posterior patterning defects is observed in the *dGe-1* mutant when one *wt* copy of *dDcp1*, *stau* or *btz*, each of which encodes a component of *osk* RNPs, is removed. Similarly, depletion of *dGe-1*, which disrupts P body assembly in *Drosophila* S2 cells, only mildly affects miRNA-mediated mRNA decay, and the knockdown of *dDcp1* or *Me31B*, both of which are involved in mRNA decapping, has no obvious effect on this process [19]. It is therefore possible that dGe-1 functions to locally concentrate the proteins essential for *osk* mRNA localization, rendering *osk* RNP transport more efficient or robust, in particular when some components are limiting. Our study suggests a novel function of dGe-1 in optimization of the process of *osk* mRNA localization.

Finally, our demonstration of the involvement of dGe-1 in *osk* mRNA localization in the *Drosophila* oocyte raises the possibility that P body components and P bodies themselves may contribute to RNA localization, a highly conserved phenomenon, in other eukaryotic cell types and organisms. In addition, the defects in Grk protein expression in *dGe-1* mutant ovaries point to a more general role of dGe-1 and presumably of P bodies in mRNA regulation.

## Materials and Methods

### Fly stocks

The following fly strains were used in this study: *w^1118^*, *YFP-dDcp1* [4], *FWS-GFP* [21], *maternal-α-tub-Gal4:VP16* [32], *P[w[+mC] = GSV2] GS5005* (Kyoto Stock Center), *oskMS2* [33], *y^1^ w^67c23^*; *P{Hsp83-MCP-RFP}12a/TM3*, *Sb^1^* (Bloomington Stock Center); *UASp-oskBoxB*, *UASp-* λ*N-GFP* (Fan and Ephrussi, unpublished data), *stau^D3^/CyO* [30], *dDcp1^b53^/CyO* [4], *khc^27^/Cyo* [28], *btz^2^/TM3* [29]. GLC were generated as described [20]. For transgenic rescue, a P element-based transgene expressing *dGe-1-B* cDNA (see below) was crossed into the *dGe-1^Δ5^* mutant background.

### Generation of *dGe-1* deletion alleles by imprecise excision

The P element bearing the mini-white gene in the fly stock *GS5005* was remobilized by imprecise excision. Candidate progeny recognized by their white eye-color were subjected to single fly PCR analysis [34] with three sets of primers against the genomic region of the *dGe-1* locus (dGe-1-806F/dGe-1-1302R, dGe-1-806F/dGe-1-1676R and dGe-1-806F/dGe-1-2626R, Table S1). Another two pairs of primers (P5′/dGe-1-2626R and P3′/dGe-1-806F, Table S1) were used to check for the presence of the P element. Finally, the extent of the deleted region in the *dGe-1* mutants was determined by sequencing. Genomic analysis of one of these, *dGe-1^Δ5^*, revealed a 681 bp-long deletion covering most of the *dGe-1* 5′ UTR, the putative *dGe-1* promoter and a small part of 3′UTR of the upstream gene, *CG6192*, and a 30 bp insertion of unknown origin in this region (Figure 1A and unpublished data).

### Generation of transgenic flies

A full-length *dGe-1-B* cDNA was generated by PCR using the dGe-1-cDNA-F/dGe-1-cDNA-R primer pair (Table S1) and EST clone LD32717 as a template. The NotI-XbaI PCR fragment was ligated to a NotI-XbaI digested pCasper4-tub vector (gift of S. Cohen). The resulting transgene was used for generation of *dGe-1* rescued transgenic flies, which thus express the *dGe-1-B* cDNA under control of the *tub* promoter.

To obtain *UASp:HA-dGe-1* transgenic flies, the *dGe-1* coding region was amplified by PCR using the EST clone LD32717 as a template and the primer set, dGe-1-gateway-F and dGe-1-gateway-R (Table S1). The amplified fragment was cloned into the UASp-based destination vector pPFHW (The *Drosophila* Gateway Vector Collection), using Gateway Technology (Invitrogen). Cloning into pPFHW results in addition of 3 Flag and 3 HA tags at the N-terminus of the insert.

### Antibody generation and western blotting

Rabbit polyclonal anti-dGe-1 antibodies were raised against a C-terminal dGe-1 peptide (amino acids 1220–1354) as described [6].

The following primary antibodies were used for Western Blot analysis: rabbit anti-dGe-1 (1∶500), rat anti-dGe-1 (1∶1,000, gift of E. Izaurralde), rat anti-Cup (1∶500) and mouse anti-Me31B (1∶1,000) (gifts of A. Nakamura), rabbit anti-Khc (1∶50,000, Cytoskeleton), rabbit anti-GFP (1∶50,000, Torrey Pines), rabbit anti-Exu (1∶20,000, gift of P. Macdonald), mouse anti-Tub (1∶10,000, DM1A, Sigma), mouse anti-HA (1∶1,000, HA.11, Convance), rat anti-Grk (1∶2,000, gift of T. Schupbach) and rabbit anti-Osk (1∶2,000).

Ovary and 0–2 h embryo extracts were prepared as described [28], [35].

### Immunoprecipitation

Dissected ovaries were homogenized in DXB-150 buffer, and centrifuged at 10,000 *g* for 10 min at 4°C [23]. The supernatant was incubated with appropriate antibodies. For immunoprecipitation of GFP- or YFP-tagged proteins, 7 µl of rabbit anti-GFP antibody (Torrey Pines) was used. For immunoprecipitation of endogeneous dGe-1 protein, 10 µl of rabbit anti-dGe-1 antibody were coupled to 25 µl (50% slurry) protein A sepharose beads (GE healthcare) at 4°C overnight on a head-over-tail rotor; the antibody-coupled beads were either incubated or not with dGe-1 peptide (also used to generate dGe-1 antibodies) [6] for 1 h at 4°C; extract was then added and incubated for 2 h at 4°C. To limit unspecific retention on the beads, the matrix was preincubated in 500 µl of blocking buffer (20 mM Hepes-KOH pH 6.95, 550 mM NaCl, 0.1 µg/µl glycogen, 0.1 µg/µl tRNA, 1 µg/µl BSA, 0.1% NP-40) for 16 h at 4°C and resuspended in DXB-150 buffer.

After immunoprecipitation, the beads were washed 6 times for 10 min with 500 µl DXB-150 buffer. For Western blot analysis, the bound proteins were boiled in 2× SDS sample buffer for 10 min at 95°C. For RT-PCR analysis, bound RNAs were extracted with Trizol (Invitrogen), and dissolved in 15 µl H_2_O.

For the RNAse A sensitivity assay, RNAse A was added to ovarian extracts at a concentration of 0.33 µg/µl prior to immunoprecipitation.

### RT-PCR

Total ovarian RNA was extracted and cDNA synthesis was performed as previously described [36]. *dGe-1-A* and *dGe-1-B* transcripts were amplified (25 PCR cycles) using the dGe-1-1166F/dGe-1-1420R primer set (Table S1). For evaluation of the relative amounts of *dGe-1* transcripts, three sets of primers, dGe-1coding-502F/dGe-1coding-899R, dGe-1coding-2037F/dGe-1coding-2352R and dGe-1coding-3070F/dGe-1coding-3345R, were used (Table S1). As a control, *rp49* was detected using rp49F/rp49R primers (Table S1).

For evaluation of mRNA amounts in dGe-1 immunoprecipitates, 5 µl RNA were used for cDNA synthesis using the Superscript III First strand System (Invitrogen), following the manufacturer's instructions. Subsequently, 3 µl of cDNA were used in a 10 µl standard PCR reaction using specific primers for *osk*, *grk*, *rp49*, *tub* and *bcd* (Table S1).

### Immunostaining and FISH

Ovaries were dissected in cold PBS and processed for immunostaining essentially as previously described [37], using the following antibodies: rat anti-Stau (1∶2,000) [38], rabbit anti-Me31B (1∶4,000), rabbit anti-Tral (1∶1,500), and mouse monoclonal anti-Cup (1∶1,000) (gifts of A. Nakamura), rat anti-dGe-1 (1∶1,000, gift of E. Izaurralde), purified rabbit anti-dGe-1 (1∶100), mouse monoclonal anti-Grk 1D12 (1∶200, DHSB), and mouse monoclonal anti-HA (1∶1,000, HA11, Convance). FISH coupled with immunodetection was performed using a rabbit anti-Osk antibody (1∶3,000) and a digoxigenin-labeled *osk* antisense probe (at a final concentration of 0.5 ng/µl).

Confocal pictures were taken using an oil-immersion 40× objective on a Leica SP2 confocal microscope and visualized using Leica Confocal Software.

### Cuticle preparation

Cuticle preparation was performed as described [39].

### Ovary extract preparation for sucrose gradient and RNA analysis

Ovaries were dissected in PBS containing 0.1% Triton X-100, washed twice in 1 ml of SB buffer (50 mM Tris-HCl pH 7.5, 250 mM KCl, 2.5 mM MgCl_2_, 0.1% Triton X-100, 2 mM DTT, 50 U/ml RNAse inhibitor, 5 mg/ml Heparin, protease inhibitors), resuspended in 200 µl of SB buffer, and homogenized. The extract was centrifuged at 16,110 *g* for 10 min at 4°C. The supernatant (180 µl) was collected, supplemented with 2 µl Tris-HCl 1M pH 7.5, 2 µl of SB1 buffer 10× (500 mM Tris-HCl pH 7.5, 2.5 M KCl, 25 mM MgCl_2_), 12.6 µl of H_2_O and 5.4 µl of cycloheximide 0.1 M and incubated for 30 min at 30°C before sucrose gradient centrifugation.

### Sucrose gradient centrifugation and RNA analysis

Typically, 200 µl of extract was layered on a 5.2 ml linear 10–45% sucrose gradients in 50 mM Tris-HCl pH 7.5, 250 mM KCl, 2.5 mM MgCl_2_ and spun in a Beckman SW60 Ti rotor at 50,000 rpm for 40 min at 4°C. 18 fractions were collected from top to bottom, using a Biocomp fractionator. The last fractions were omitted from analysis. RNA was isolated with Trizol (Invitrogen) and RNA levels determined by RNase protection assay (Ambion, Inc.) according to the manufacturer's instructions.

The samples were then subjected to electrophoresis on a 5% polyacrylamide/8M urea gel for 2 h at 90W. Data were visualized using Fuji phosphorimager FLA 2100 and quantified using MultiGauge software.

## Supporting Information

Figure S1 ***dGe-1*** **transcripts are expressed in the** ***Drosophila*** **germline and dramatically reduced in** ***dGe-1^Δ5^ GLC*** **ovaries.** (A) RT-PCR amplification of *dGe-1-A* and *dGe-1-B* mRNAs from a *wt* ovarian extract using a pair of primers flanking the alternatively spliced intron of *dGe-1*. The *dGe-1-A* and *dGe-1-B* amplified PCR fragments are of 171 and 254 bp, respectively. (B) The amount of *dGe-1* mRNA is severely reduced in *dGe-1^Δ5^ GLC* ovaries. RT-PCR amplification of *dGe-1* transcripts from *wt* and *dGe-1^Δ5^ GLC* ovarian extracts using three pairs of primers targeting different regions of the *dGe-1* transcripts (blue, red, and green primers, see Figure 1A). *rp49* serves as a loading control.
(TIF)

Figure S2 **dGe-1 affects Grk protein expression.** (A) Immunoprecipitation of endogenous dGe-1 from *wt* ovary extract using rabbit anti-dGe1 antibodies either pre-incubated or not with the dGe-1 peptide used for generation of the dGe-1 antibodies in this study. Rabbit IgG antibody serves as a non-specific immunoprecipitation control. Total RNA extracted from the bound fractions was subjected to semi-quantitative RT-PCR (25 cycles). *osk*, *grk*, *bcd*, *rp49* and *tub* RNAs were analyzed. (B–C) Distribution of *bcd* mRNA in *wt* (B) and *dGe-1^Δ5^ GLC* (C) oocytes (in red). DNA stained with DAPI (blue). (D,F) Distribution of *grk* mRNA in *wt* (D) and *dGe-1^Δ5^ GLC* (F) oocytes, detected by FISH (in red). DNA stained with DAPI (blue). (E,G) Distribution of Grk protein (green) in *wt* (E) and *dGe-1^Δ5^ GLC* (G) oocytes. DNA stained with DAPI (blue). (H) Western blot analysis of ovary and embryo extracts from *wt* (first lane), *dGe-1^Δ5^ GLC* (second lane), *dGe-1 R* (third lane) females probed with rat anti-Grk and mouse anti-Tub antibodies. The band around 50 kDa specific to Grk protein is indicated. Tub serves as a loading control. Bar, 50 µm.
(TIF)

Figure S3 ***dGe-1*** **cooperates with** ***stau*** **and** ***dDcp1*** **in** ***osk*** **mRNA localization at S9.** (A) Quantification (%) of the different *osk* mRNA localization phenotypes in S9 egg-chambers of different genetic backgrounds. Normal and abnormal *osk* mRNA localization are represented by dark and grey bars, respectively. *n* represents the number of embryos analyzed. (B–D) Distribution of *osk* mRNA in *wt* (B), *dGe-1^Δ5^ GLC* (C) and *dGe-1 R* (D) oocytes (in red). DNA stained with DAPI (blue). (E) Western blot analysis of ovarian extracts from *wt* (first lane) or *dGe-1^Δ5^ GLC* (second lane) females probed with rabbit anti-Osk and mouse anti-Tub antibodies. Tub serves as a loading control. Bar, 25 µm.
(TIF)

Table S1 **List of primers used for cloning and RT-PCR analysis.**
(DOC)
