# Supplementary figures and images for: Drosophila Ge-1 Promotes P Body Formation and oskar mRNA Localization
Source: PLoS One. 2011 May 31;6(5):e20612. doi: 10.1371/journal.pone.0020612 (PMC3105097; doi:10.1371/journal.pone.0020612)

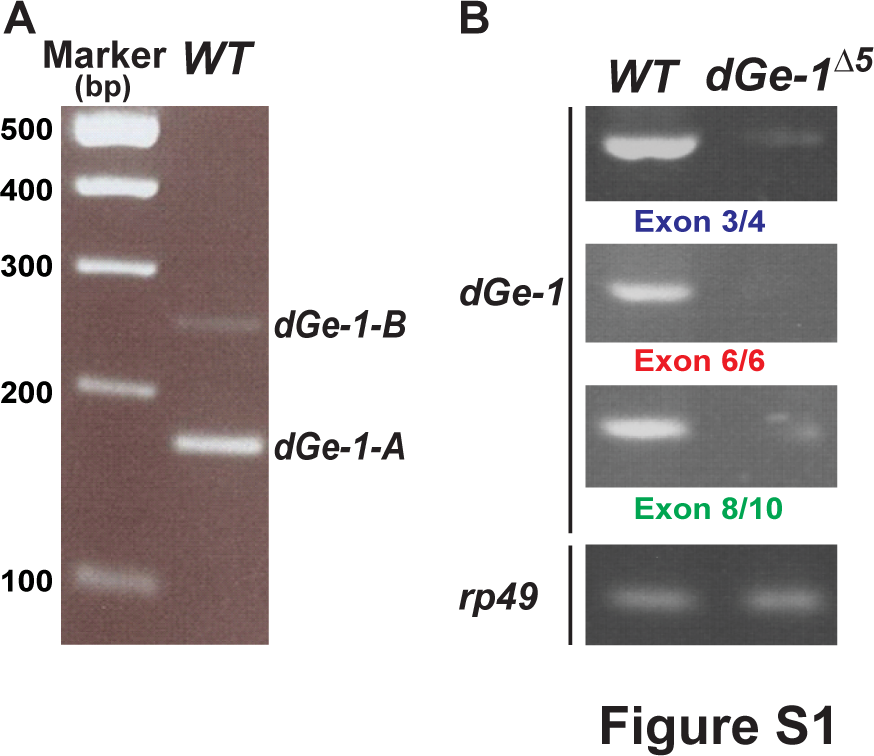

Supplement: Figure S1 — dGe-1 transcripts are expressed in the Drosophila germline and dramatically reduced in dGe-1Δ5 GLC ovaries. (A) RT-PCR amplification of dGe-1-A and dGe-1-B mRNAs from a wt ovarian extract using a pair of primers flanking the alternatively spliced intron of dGe-1. The dGe-1-A and dGe-1-B amplified PCR fragments are of 171 and 254 bp, respectively. (B) The amount of dGe-1 mRNA is severely reduced in dGe-1Δ5 GLC ovaries. RT-PCR amplification of dGe-1 transcripts from wt and dGe-1Δ5 GLC ovarian extracts using three pairs of primers targeting different regions of the dGe-1 transcripts (blue, red, and green primers, see Figure 1A). rp49 serves as a loading control. (TIF) [file pone.0020612.s001.tif]

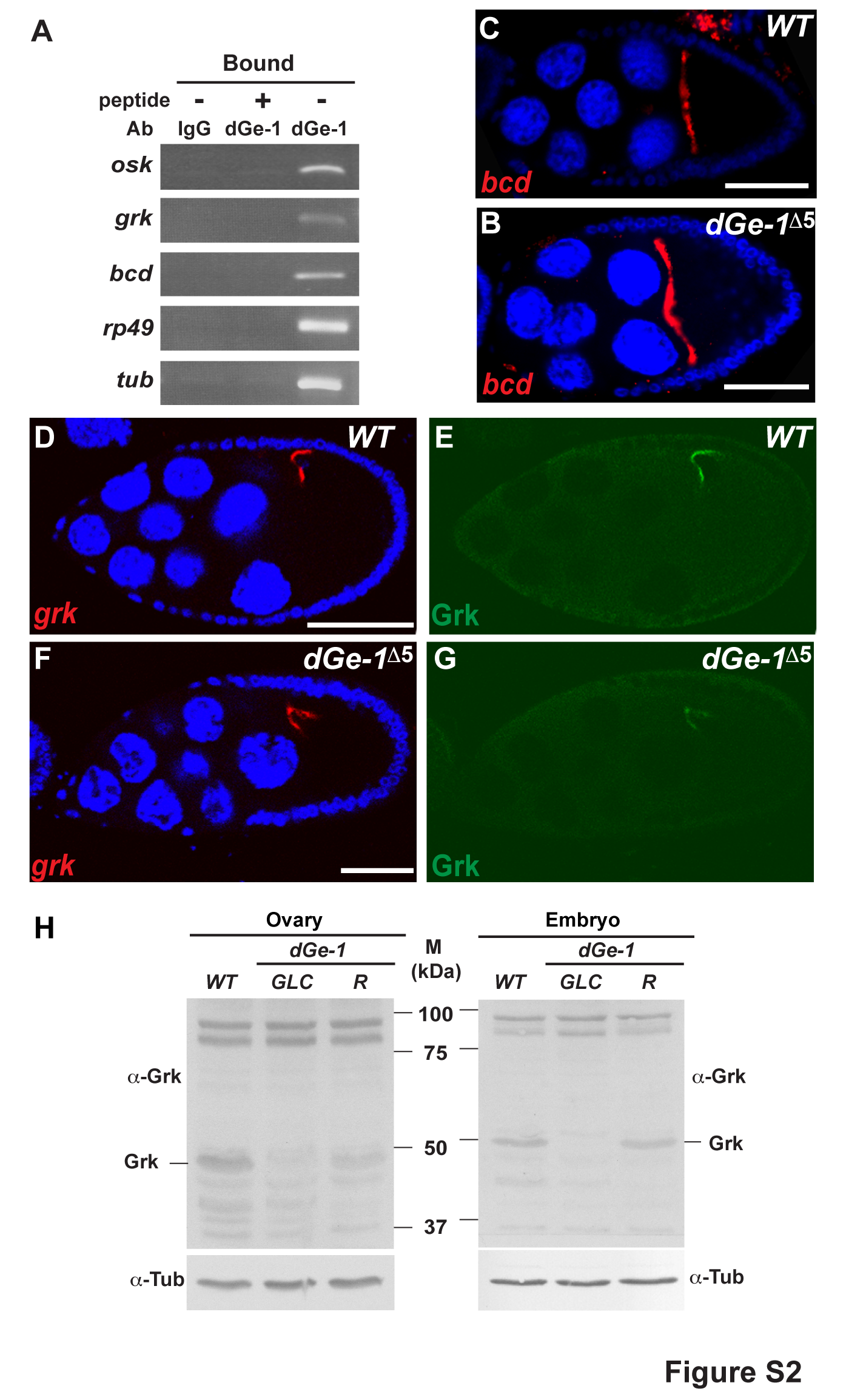

Supplement: Figure S2 — dGe-1 affects Grk protein expression. (A) Immunoprecipitation of endogenous dGe-1 from wt ovary extract using rabbit anti-dGe1 antibodies either pre-incubated or not with the dGe-1 peptide used for generation of the dGe-1 antibodies in this study. Rabbit IgG antibody serves as a non-specific immunoprecipitation control. Total RNA extracted from the bound fractions was subjected to semi-quantitative RT-PCR (25 cycles). osk, grk, bcd, rp49 and tub RNAs were analyzed. (B–C) Distribution of bcd mRNA in wt (B) and dGe-1Δ5 GLC (C) oocytes (in red). DNA stained with DAPI (blue). (D,F) Distribution of grk mRNA in wt (D) and dGe-1Δ5 GLC (F) oocytes, detected by FISH (in red). DNA stained with DAPI (blue). (E,G) Distribution of Grk protein (green) in wt (E) and dGe-1Δ5 GLC (G) oocytes. DNA stained with DAPI (blue). (H) Western blot analysis of ovary and embryo extracts from wt (first lane), dGe-1Δ5 GLC (second lane), dGe-1 R (third lane) females probed with rat anti-Grk and mouse anti-Tub antibodies. The band around 50 kDa specific to Grk protein is indicated. Tub serves as a loading control. Bar, 50 µm. (TIF) [file pone.0020612.s002.tif]

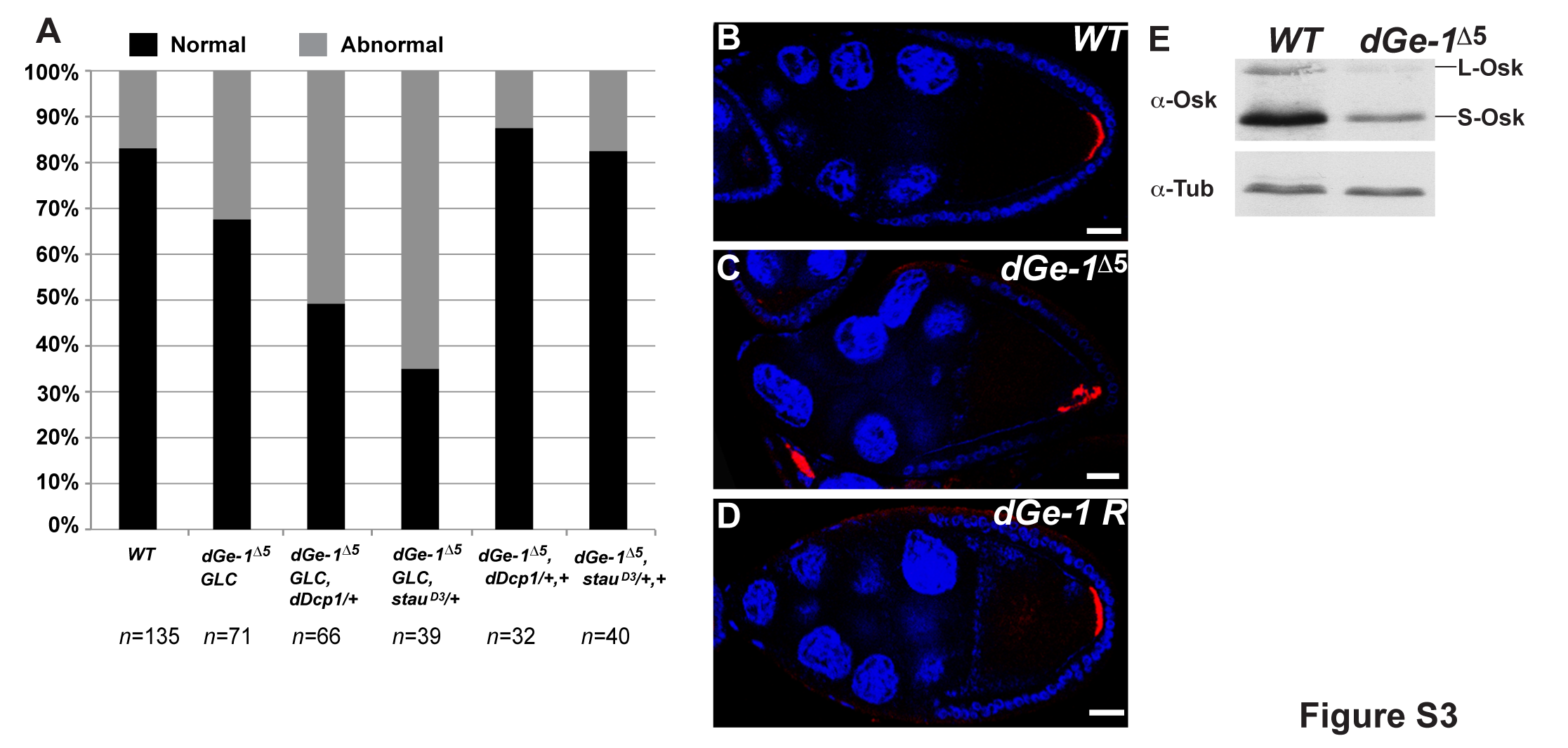

Supplement: Figure S3 — dGe-1 cooperates with stau and dDcp1 in osk mRNA localization at S9. (A) Quantification (%) of the different osk mRNA localization phenotypes in S9 egg-chambers of different genetic backgrounds. Normal and abnormal osk mRNA localization are represented by dark and grey bars, respectively. n represents the number of embryos analyzed. (B–D) Distribution of osk mRNA in wt (B), dGe-1Δ5 GLC (C) and dGe-1 R (D) oocytes (in red). DNA stained with DAPI (blue). (E) Western blot analysis of ovarian extracts from wt (first lane) or dGe-1Δ5 GLC (second lane) females probed with rabbit anti-Osk and mouse anti-Tub antibodies. Tub serves as a loading control. Bar, 25 µm. (TIF) [file pone.0020612.s003.tif]
