# Supplementary material for: Drosophila Ge-1 Promotes P Body Formation and oskar mRNA Localization
Source: PLoS One. 2011 May 31;6(5):e20612. doi: 10.1371/journal.pone.0020612 (PMC3105097; doi:10.1371/journal.pone.0020612)
Supplement: Table S1 — List of primers used for cloning and RT-PCR analysis. (DOC) [file pone.0020612.s004.doc]

| Name of the primer | Primer Sequence (5’ to 3’) | Used for |
| --- | --- | --- |
| dGe-1-806F | CCACCACACACAATCACAC | Single fly PCR |
| dGe-1-1302R | CCTATCAATACGCAGCAGTC | Single fly PCR |
| dGe-1-1676R | ACGCACATGACTTCTATTTCC | Single fly PCR |
| dGe-1-2626R | ATTAGCACAAACGAACCCC | Single fly PCR |
| P3’ | ACTCAATACGACACTCAGAATACT | Single fly PCR |
| P5’ | CACCCAAGGCTCTGCTCCCACAAT | Single fly PCR |
| dGe-1-cDNA-F | GAGCGGCCGCACACGCCGCTACACACCTCTA  *^NotI^* | Cloning of *dGe-1-B* cDNA into pCasper4-tub vector |
| dGe-1-cDNA-R | GGTCTAGAAAAATAATAAAAAAATATATTGCA  *^XbaI^* | Cloning of *dGe-1-B* cDNA into pCasper4-tub vector |
| dGe-1-gateway-F | CACCATGTTAATCGCGCTCTTCGCGC | Cloning of dGe-1 cDNA into *UASp-HA-dGe-1* |
| dGe-1-gateway-R | TTTTAGCTGATCGCGGTACGTTAT | Cloning of dGe-1 cDNA into *UASp-HA-dGe-1* |
| dGe-1-1166F | TTTACGAGAAGCAGCCAAC | RT-PCR *dGe-1- A and B* |
| dGe-1-1420R | AGAGCGCGATTAACATTGAC | RT-PCR *dGe-1*-*A and B* |
| dGe-1coding-502F | GCATGGTGCGCGTATGCAAC | RT-PCR *dGe-1* |
| dGe-1coding-899R | GCTGCTGGTTGGATCTTGCC | RT-PCR *dGe-1* |
| dGe-1coding-2037F | ACCACCAGCGGTCAGGATAG | RT-PCR *dGe-1* |
| dGe-1coding-2352R | CTCTGCTTCGTAGGCATCGG | RT-PCR *dGe-1* |
| dGe-1coding-3070F | TCAACATGGAACTGCAGCGCC | RT-PCR *dGe-1* |
| dGe-1coding-3345R | TATGCCCACGCTGAAAGCGTC | RT-PCR *dGe-1* |
| rp49F | GCTAAGCTGTCGCACAAA | RT-PCR *rp49* |
| rp49R | TCCGGTGGGCAGCATGTG | RT-PCR *rp49* |
| osk2460F | GGCCGTAATGAAAATCGACT | RT-PCR *oskar* |
| osk2683R | TCTACACTGTGCTACAAACAAG | RT-PCR *oskar* |
| bcdF | GACCTTGCGCCATCGCCGTT | RT-PCR *bicoid* |
| bcdR | ACCCTTCAAAGGCTCCAAGAT | RT-PCR *bicoid* |
| tub67cF | GGCAGCCTGAAGACCAAGGAGGAG | RT-PCR *tubulin* |
| tub67cR | CACTGCTCTGCGATCTTCTGC | RT-PCR *tubulin* |
| grk F | AAGCAGCAGCAACTACACC | RT-PCR *gurken* |
| grk R | AGCAAATACTAACTTGTGCGTC | RT-PCR *gurken* |

**Supplementary Table S1:** **List of primers used for cloning and RT-PCR analysis.**
